# Effects of the Specific Eye Fixation Training on Fine Visuomotor Coordination in Children with Attention Deficit Hyperactivity Disorder

**DOI:** 10.3390/children10101648

**Published:** 2023-10-03

**Authors:** Ludvík Valtr, Rudolf Psotta, Daniel Dostál

**Affiliations:** 1 Department of Natural Sciences in Kinanthropology, Faculty of Physical Culture, Palacký University Olomouc, 771 11 Olomouc, Czech Republic; psotta@palestra.cz; 2 Department of Wellness and Nutrition, College of Physical Education and Sport PALESTRA, 197 00 Prague, Czech Republic; 3 Department of Psychology, Faculty of Arts, Palacký University Olomouc, 779 00 Olomouc, Czech Republic; daniel.dostal@upol.cz

**Keywords:** manual dexterity, ADHD, intervention, attention, children

## Abstract

This study investigated the effects of quiet eye training (QET) on the neuropsychological functioning and fine motor performance of children with attention deficits. The participant cohort consisted of 106 children with attention deficit hyperactivity disorder (ADHD) between the ages of 8 and 12 years. The children were assigned to either the QET group (*n* = 54) or the control group (*n* = 52). The QET group went through a 5-week intervention in which the performance of blocks of targeting tasks was preceded by watching split-screen video footage featuring the gaze and body movements of a skilled model performing a throwing motion. Both groups underwent pre-test and post-test assessments, which included the reaction test of alertness, go/no-go inhibition test, and motor performance series test. The QET group demonstrated significant improvements in attentional engagement, inhibitory control, and fine motor skills, which require precise and fast visuomotor coordination. These results highlight the potential benefits of QET intervention in ameliorating attention deficits and enhancing fine aiming motor skills in children with ADHD. However, task specificity was evident, indicating that the intervention effects were most pronounced for the hand fine motor aiming tasks requiring both precision and speed.

## 1. Introduction

Children with attention deficit hyperactivity disorder (ADHD) often have difficulties with daily living activities that require body coordination, although motor difficulties are not diagnostic features of this neurodevelopmental disorder [1]. Motor deficits can be found across different motor areas in children with ADHD; however, atypical fine motor control is often reported as a more stable trait [2]. More specifically, studies involving individuals with ADHD have reported that they perform manual dexterity tasks less well [3] and demonstrate trouble grasping and manipulating objects [4], difficulty writing clearly [5], and poor fine motor precision and integration [6]. In addition, children with ADHD require more time to prepare their movements and are more dependent on online movement control, and their movements are characterised by higher variability, meaning that they have difficulty parameterising movements consistently [2].

One of the two hypotheses that explains the lower motor skill performance among children with ADHD is that it may be caused by ADHD symptoms, such as impaired attention [2] and poor behavioural inhibition [7].

First, impaired visual-focused attention and deficit inhibition control in children with ADHD are also indicated by difficulties in maintaining eye fixations [8] and inhibition of automatic saccades [9] in tasks demanding eye fixations and anti-saccades, respectively. Problems with focused and sustained visual attention can disrupt the visual information capture and processing necessary for motor programming [10], especially for visually guided tasks [11]. Fabio et al. [12] demonstrated that children with ADHD have visual–motor attention impairments, which refer not only to visual information capture and processing but also to motor control.

Second, motor problems in children with ADHD may be secondary to comorbid developmental coordination disorders (DCD) [2]. The mechanisms underlying DCD are related to deficits in predictive (feed-forward) control, including motor programming and online motor control [13]. Up to 50% of children with ADHD are reported to have reduced motor competence [2]. However, evidence of the relationship between inattention and motor problems has been supported by studies in which medication (methylphenidate) led to an improvement in attention that, in turn, improved motor performance [2]. The linkage between impaired attention and motor difficulties may also be supported by neuroimaging studies that have reported abnormalities in the brain regions associated with motor function in individuals with ADHD [14]. Specifically, the functional and structural neural circuits associated with sensory and higher-order cognitive functions are altered in children with ADHD [15]. The brain areas that play roles in attention, thought, and motor planning, such as the prefrontal cortex, cerebellum, and caudate [16], have been reported to show delayed activity and development in individuals with ADHD [17].

One possible way to ameliorate impaired fine motor skills in children with ADHD is through an intervention that aims to improve motor tasks that demand attention [18]. Chang et al. [19] indicated an improvement in handwriting in children with ADHD who participated in table tennis training with a focus on short- and long-term visuomotor control. In a study by Saemi et al. [20], an external focus of attention, specifically concentration on the landing location of the ball at the target, was shown to be beneficial for learning to throw the ball at the target in children with ADHD. Gaze-contingent eye-tracking training has been implemented to enable children to execute complex cognitive tasks to improve sustained attention and inhibition (for a review, see [21]).

The specific instructional design called quiet eye training (QET), developed by Vickers [22] for the improvement of targeting and interceptive motor skills, has been hypothesised to stimulate improvement in attentional control, allowing for greater cognitive effort to be devoted to the task and, as such, improving motor learning and visuomotor skill performance [23]. QET consists of performing a goal-directed visuomotor task, during which individuals are provided with instructions and video demonstrations of a skilled model of eye fixations synchronised with a skilled model of a motor action to optimise their focused attention and gaze and, in turn, refine visuomotor control [22,24]. QET is based on the quiet eye theory, which holds that a final eye fixation on a relevant object––referred to as the quiet eye––before the initiation of a body movement is critical for the planning and programming of motor action [22].

To the best of our knowledge, only five studies on the efficacy of QET have been published. The studies have suggested that QET can lead to optimised gaze control, specifically earlier onset and/or timely prolonged quiet eye to a relevant location, indicating positive changes in attention control, and concurrently improve motor skills, such as catching and throwing, in typically developing children and children with developmental coordination disorder (DCD) [25,26].

Based on the findings of the recent studies on QET mentioned above and the knowledge of the functional linkage of gaze control, motor control, and attention, we hypothesised that a QET-based intervention would be beneficial for the improvement of fine motor dexterity in children with ADHD via the stimulation of attention and goal-directed gazing while practising in targeting tasks. This study aimed to test this hypothesis.

## 2. Materials and Methods

### 2.1. Participants

A total of 106 children aged 8–12 years who met the diagnostic criteria for the inattentive subtype (*n* = 91) or the combined subtype of ADHD (*n* = 15) according to the Diagnostic and Statistical Manual (DSM-5) [1] participated in the training intervention study. All children were from 12 Czech mainstream public schools where the training interventions and measurements were conducted. Children with psychotic and conduct disorders and physical, visual, hearing, or neurological impairments were not recruited for this study. The children from the schools were randomly assigned to the QET-based intervention group (*n* = 54, age 10.3 ± 1.1 years) or the control (CON) group (*n* = 52, age 10.7 ± 0.9 years). No significant differences were found between the groups in terms of age (*p* = 0.106), percentage of boys (*p* = 0.736), ADHD subtype (*p* = 0.427), and medication (*p* = 0.454) (Table 1). Written informed consent was obtained from the legal guardians of the children who participated in the study. The participants and their legal guardians were not aware of the aim of the study; the legal guardians of the children were only told that training lessons would involve different motor skill activities to ameliorate impaired attention. Ethical approval for the study was obtained from the Ethical Committee of the Palacký University in Olomouc, Faculty of Physical Culture, the code of the approval FTK 31/2018. This study was performed in accordance with the Declaration of Helsinki, 1975.

### 2.2. Study Design

The study was designed as a blinded controlled trial that involved a 5-week training phase. The pre- and post-measurement phases were conducted one week before the first training lesson and one week after the last training lesson for the QET group. Each participant in the CON group performed the post-measurements six weeks after the pre-measurements without participation in QET.

### 2.3. Pre- and Post-Measurement Phases

The participants in both groups underwent identical pre-test and post-test sessions that involved a neuropsychological assessment with the reaction test of alertness (RTA), go/no-go inhibition (GNG) test, and motor performance series (MLS). The three tests were performed in a counterbalanced order within each group.

#### 2.3.1. Reaction Test of Alertness

The standardised, computerised RTA of the Vienna Test System version 31 (Schuhfried GmbH, Mödling, Austria) was used. This test consisted of 28 simple reaction trials with a stimulus in the form of a yellow circle (diameter of 3 cm) followed by a second set of 28 trials that involved an acoustic warning signal preceding the stimulus. The study’s aim was to assess tonic (intrinsic) attention; therefore, the first set of non-signal trials was employed. In this test, the stimulus was presented in the centre of a black background on a computer screen. From the initial position of the index finger of the preferred hand on the home button (diameter of 2 cm), the participant responded to the stimulus as quickly as possible by pressing the rectangular black (response) button (5.5 × 1.5 cm) on the operating panel. After responding to the stimulus, the participant placed their index finger back on the home button. The home button was located below the response button with the near edges at a distance of 45 mm. The reaction time (RT) was measured as the interval between the moment of appearance of the stimulus and the moment the index finger left the home button. The display of the stimulus remained until a response was detected for a maximum of 1000 ms. The stimuli were presented at inter-stimulus intervals (ISI) from 2500 ms to 6500 ms.

The following variables were assessed: mean RT as a measure of tonic attention (MRT), intraindividual standard deviation of RTs (SDRT) as a measure of arousal regulation variability, and the number of correct responses (corrR) and incorrect responses (incorrR). Incorrect responses were identified using the VTS software. The reliability of the RTs measured in the non-signal trials of the RTA was reported to be excellent (*r* = 0.965) [27].

#### 2.3.2. Go/No-Go (GNG) Inhibition Test

The GNG PsyToolkit test was used to assess inhibitory control in the participants. The participants had to respond as quickly as possible to a go stimulus (green oval, width 5 cm, height 2 cm) by pressing the spacebar and refraining from this response in the case of a no-go stimulus (red oval, equal dimensions as the green oval). The GNG stimuli were presented individually at the centre of a black background on a computer screen for 500 ms at an inter-stimulus interval of 2000–4000 ms at a ratio of GNG stimuli (go/stop trials) of 80/20 and provided at random. The participants were initially presented with 20 practice stimuli, and then, they completed 100 trials. The duration of the test, including instructions and practice, was 10 min.

The following variables were assessed as measures of inhibition: mean reaction time to go stimuli (MRT_GNG_); intraindividual standard deviation of RTs to go stimuli (SDRT_GNG_); omission, that is, the number of no responses during go trials; and commission, that is, the number of responses given during no-go trials. Only correct responses to go-stimuli with RT ≥ 100 ms were included to calculate MRT_GNG_ and SDRT_GNG_. RTs of <100 ms in the no-go trials did not consider commission errors.

A simple GNG RT task was used to measure the children’s response inhibition [7]. Moderate-to-high reliability of this type of GNG inhibition test has been reported in previous studies [28,29]. The measurements of the GNG test were evaluated using a reference range of 367–431 ms for the MRT_GNG_, 3–15 for the number of omissions, and 0–6 for the number of commissions. These reference ranges were identified in our pilot study with typically developing children aged 10–11 years (*n* = 62), according to the formula 95% CI = mean ± *t*_0.975,*n*−1_·√(*n* + 1)/*n*·SD.

#### 2.3.3. The Motor Performance Series (MLS) Test

The short form (S3) of the MLS of the Vienna Test System (Schuhfried GmbH, Mödling, Austria) was used to assess the fine motor skills (manual dexterity). The MLS test consists of ten subtests with static and dynamic movements of the fingers, hand, and arm performed by the individual on a work panel (dimensions 30 × 30 × 1.5 cm) and a contact pen. The participants first performed five subtests with the dominant hand followed by identical subtests with the non-dominant hand in the following order:

Steadiness: The task was to hold a contact pen in a vertical static position in the slot (a diameter of 5.8 mm) with the least number of contacts of the pen with an inner side or the bottom of the slot for 32 s. The score used was the total time of error, which is the sum of the duration of all contacts of the pen with the inner side and bottom of the slot. This task measures the static positions of the hand and arm, which are indicators of hand tremors [15].

Line tracing: The task was to guide an electric pen perpendicular to the plane of the work panel along a groove (width of 5 mm) that changed direction as quickly and precisely as possible without touching the pen on the wall of the groove. The scores used were the time of tracing, that is, the total time required to perform the task and the total time of errors (an error = touching the pen to the wall of the groove). Line tracing measures the ability to perform precise arm and hand movements [15].

Aiming: The task was to touch 20 gold plates (diameter of 5 mm, 4 mm apart) located consecutively in a row from right to left (for the right hand) or from left to right (for the left hand) as quickly as possible without missing the plates. The scores, that is, the number of hits, total time of errors (error = a touch outside the plate), and time needed to perform the task were included in further analysis. This task is a measure of the precision and speed of hand and arm movements coordinated to the locations of the targets [15].

Tapping: The task was to tap an electronic pen on a contact square plate (40 × 40 mm) as frequently as possible for 32 s. This was the only subtest in which the participants were allowed to rest their forearm and wrist on a work panel. The measured score was the number of hits. Tapping is an indicator of the ability to perform rapid repetitive hand movements without specific precision [15].

Inserting pins: The task was to pick up long metal pins (50 mm long, 2 mm in diameter), successively one to the other, from the pin box located 30 cm from the side edge of the work panel corresponding to a working hand and insert them into the slots of the work panel as quickly as possible. The insertions were performed successively from top to bottom. The measured score was the time required to insert the 25 pins.

The all-time scores were measured with an accuracy of 0.01 s. Accurate wording of instructions, practice, and conditions for the execution of the subtests were adhered to according to the MLS test manual [15]. The participant’s nonworking hand rested on a table adjacent to the work panel. A black or red contact pen was used to perform the subtests with the right and left hands. The total time required to perform all tests, including the instructions, was 15–20 min with a mean time of 17 min 21 s.

According to Fleishman’s factor analytic research on fine motor skills, namely aiming movement, stability of hand fidgety, tremors, precision of hand and arm movement, hand and finger dexterity, and speed of wrist and finger movements [15], the six factors of the MLS test were reported to explain more than 85% of the total variance. For the MLS subtests, a good up to excellent reliability was presented, specifically for the scores of the line tracing, aiming and tapping test–retest reliability (1 day of a test–retest interval) *r* = 0.52 up to 0.92 [15], and for the tapping Cronbach’s coefficient of consistency α = 0.94, and split-half reliability *r* = 0.84–0.92 in children aged 7 to 12 years [15].

### 2.4. QET-Based Intervention

**Figure 1:**
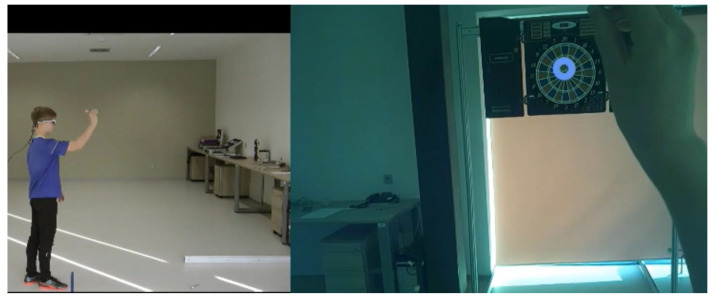
An illustration of a split-screen video used to demonstrate the dart-throwing task (the motor video on the left half and the gaze video on the right half of the screen).

Patients in the QET group underwent QET according to the protocol described by Miles et al. [25]. The QET in this study consisted of performing target tasks (Table 2). The practice of these tasks was supported by providing instructions and participant observation of split-screen video footage with the gaze and body movements of a skilled model during a throwing motion to emphasise the focus of the participant’s gaze on a target (Figure 1). The split-screen video for each task (Table 2) was created from eye-tracker records, including the location of a focal point within the visual field with a target, and from bodily movement records taken from the sagittal view of a highly skilled 12-year-old boy during the targeting task. To create eye behaviour records, the performer wore mobile SMI Eye Tracking Glasses 2w (ETG) (SMI, Teltow, Germany), which recorded eye movements (24 Hz) and eye positions (60 Hz, gaze position accuracy of 0.5°). The ETG was connected to a mobile smart recorder (a customised Samsung Galaxy S4 Smartphone). The gaze and body movement videos were synchronised and created at playback speeds of 100%, 50%, and 25% in one split-screen video using Dartfish 6.0 video analysis software (Dartfish, Fribourg, Switzerland).

The QET consisted of five 35 min training sessions, one session per week (Table 2), conducted at the school of the participant and guided by three trained gradual instructors. The sessions were conducted according to the following protocol:

Step 1: Instruction and demonstration of the task by the instructor.

Step 2: Participants watched the split-screen video on a tablet with a focus on gaze at the motor video at the three playback speeds (approximately 90 s) and, then, at the gaze video (approx. 90 s) accompanied by instructions to optimise gaze before the initiation of a throw.

Step 3: Participants summarised how to gaze during the pre-throw and throw phases.

Step 4: The participant performed 30 practice trials of a task; after each of the five trials, the instructor encouraged the participant to focus on the target while throwing.

Step 5: Participants watched the gaze video and received short instructions on the major points for optimising gaze while throwing.

Step 6: The participants performed 20 practice trials of the task. After a 4 min break, the participants performed various modifications to the task (Table 2) using the 6-step training protocol presented above.

### 2.5. Data Analysis

Non-parametric ordinal regression was used to analyse the data, owing to the diverse probability distributions of the dependent variables, which did not conform to the normal distribution and, therefore, did not satisfy the assumptions of a linear model. The skewness coefficients of the dependent variables ranged from −2.07 to 3.13 with 14 of the 24 dependent variables having an absolute skewness coefficient greater than 1. In addition, the Shapiro–Wilk test revealed a significant deviation from the normal distribution of 24 variables.

Specifically, a mixed-effects probit proportional odds model was employed. In the analysis, group (QET vs. CON), time (pre-test vs. post-test), and the interaction between these two factors were treated as fixed effects, whereas the proband was considered a random effect. The values of all the dependent variables were discretised into four ordinal categories with approximately equal numbers of data points in each category using a deterministic algorithm. The proportional odds assumption was tested for each model, and no violations were found. The ordinal package (https://CRAN.R-project.org/package=ordinal, accessed on 14 September 2023) in the R environment was used for the calculations.

One benefit of the probit model is that it allows for us to interpret the regression coefficients as effect sizes. In the probit regression, we predict the latent response variable, which is an underlying continuous variable expressed as a *z*-score. This represents the personal characteristics of the individual that we measure. For example, if the probit model is used to predict the number of errors (a variable that has been transformed into ordinal categories) and the regression coefficient for the time factor is *β* = 0.5, this means that the difference between the pre- and post-test is half the standard deviation in the probands’ tendency to make errors. The level of α = 0.05 is set for all tests.

## 3. Results

The descriptive statistics of the results of the three neuropsychological tests (median and median absolute deviation for all variables found in the pre- and post-tests in both groups) are presented in Table 3.

Table 4 summarises the differences in the latent response variable between the pre-test and post-test in the QET and control groups (see ‘β’) and also provides the results of a statistical test of whether the magnitude of change is different between the groups, that is, a test of the interaction (see ‘β’ for the interactions). The most relevant indication of the effect of the QET-based intervention is significant interactions with the regression weight differing significantly from zero (*p* < 0.05), meaning that the change in the latent response variable between the pre-test and post-test differs between the QET and CON groups.

A significant interaction time x group was revealed for the number of correct responses (corrR) in the RTA, number of omissions and commissions in the GNG inhibition test, and number of hits and time of errors in the aiming subtest performed with the right hand. Specifically, the QET group showed an increased corrR compared to no change in the CON group (*p* = 0.039). In the GNG test, the QET group demonstrated a significant reduction in both omissions and commissions compared to pre- and post-medians (from 35 to 23, *p* = 0.005, and from 7 to 4, *p* = 0.038), while the CON group showed non-significant changes in omissions (Table 3 and Table 4), resulting in significant interaction time x group, *p* = 0.004 and 0.014, respectively.

Comparing the pre- and post-values, the QET group demonstrated a significant increase in the number of hits and a reduction in the time of errors in the aiming subtest performed with the right hand (*p* = 0.001 and 0.012), while no significant changes were found in the CON group with significant interaction (*p* = 0.002 and 0.011, respectively).

## 4. Discussion

In this study, the participant cohort comprised children exhibiting pronounced attention deficits on average, as evidenced by their diminished performance on the RTA test corresponding to 16.5 ± 11.9th percentile according to the norms for RTA [27]. The RTA is frequently used to assess attentional impairments and is inherently aligned with the symptomatic criteria of ADHD. The inclusion criteria that encompassed participants with attenuated attentional faculties gained significance by considering ADHD, which is characterised by persistent patterns of inattention, hyperactivity, and impulsivity [1]. By deliberately targeting individuals with attentional insufficiency, our investigation sought to explore the potential merits of QET in mitigating fine motor skill deficits and attentional limitations frequently observed in children with ADHD [5].

Our findings indicated that the QET-based intervention had a notable impact on several aspects of neuropsychological functioning and motor performance. The reduction in both omissions and commissions during the GNG test indicated a decreased processing time and enhanced inhibitory control in the QET group. Commission errors reflect an individual’s inhibitory response ability rather than the strong activation of a prepotent response [7]. The improvement in commission errors aligns with previous studies by McLoughlin et al. [30] and Pliszka et al. [31] who highlighted the connection between attentional focus and inhibitory processes. The QET intervention appeared to facilitate a more focused attentional state that, in turn, contributed to better inhibitory control. The decrease in omission errors within the QET group might have contributed to the enhanced ability to differentiate go stimuli from no-go stimuli and faster generation of motor responses. In this study, an omission error was recorded when an individual failed to react to a go stimulus within the 500 ms timeframe, according to the GNG tests used in children with ADHD [32,33]. Therefore, the decrease in omission errors can be partly explained by the decrease in processing time to less than 500 ms during the go trials. This is in line with previous research that indicated enhancements in resistance to distractions following just three days of QE training [34,35].

Furthermore, the significant increase in the number of correct responses in the RTA group suggests that the QET group demonstrated improved attentional engagement, alertness, and potential fine motor precision compared to the CON group. A correct response in the RTA was defined as releasing the rest button and pressing the reaction button on a critical stimulus [27]. However, there was more than one button on the control panel, and another button with the same shape and size but different colours was placed above the reaction button. If the button was pressed instead of the reaction button, no correct response was recorded. Therefore, by observing an increased number of corrR, it is plausible to infer an enhancement in the participants’ fine motor abilities, particularly fine visuomotor accuracy, which manifests in the participants precisely guiding the movement with their finger to the required position. This outcome aligns with the premise that the QET intervention could contribute to the amelioration of precise motor control, substantiating prior research that underscored the relationship between prolonged visual fixation, sustained attention, and augmented motor skills [36,37]. The pronounced improvement in accurate responses not only signifies the efficacy of QET but also underscores its potential in cultivating finer aspects of motor proficiency. This finding reinforces the contention that modulation of visual attention facilitated with QET can be a pivotal factor in refining the intricate interplay between visual perception and motor execution.

The findings discussed in the preceding paragraph are further supported by the improvement in the performance of the MLS aiming task, as indicated by an increase in the number of hits and a decrease in the number of errors. The task demands precise and rapid hand movements aligned with the enhanced fine motor accuracy observed in the QET group. The participants’ ability to achieve better accuracy and control during the MSL task after the QET intervention substantiates the notion that the intervention not only positively influenced attention and inhibitory control but also contributed to refining the precision of the visually guided reaching skill performed in a time-restricted condition. This aligns with previous research that emphasised the interconnection among attention, inhibitory control, and motor skills [23,36].

However, it is noteworthy that the effects of the QET intervention were predominantly observable in the specific context of the aiming fine motor task, which required rapid and precise hand movements. While the participants demonstrated an enhancement in fine visuomotor accuracy under time-restricted conditions in the aiming task, no improvement was observed in the performance of the other MLS tasks. The factor specificity of the MLS tasks was apparent. According to the results of the factor analysis of the MLS tasks in an adult sample [27], performance of the aiming task with the right hand showed a factor loading on factor 1, specifically the number of errors, 0.87, and number of hits, 0.59, while factor loadings of the scores of the other MLS tasks on factor 1 were entirely negligible in the range of 0.0–0.12. Thus, the other tasks performed by the participants in this study potentially involved different visuomotor functions and did not exhibit the same level of improvement, indicating a degree of task specificity in the observed effects. This can be explained by considering the intricate and multifaceted nature of motor skills. Fine motor skills, such as those that require precise and rapid movements in an aiming task, constitute a distinct subset of a broader spectrum of motor abilities. Different tasks involve different neural circuits [38]. Therefore, it is reasonable to infer that the QET intervention likely influenced only neural circuits and cognitive processes that are intricately tied to inhibitory control and fine motor tasks that require swift precise visuomotor coordination. Other tasks within the MSL domain may engage different neural substrates or cognitive mechanisms, explaining the absence of widespread improvement across all tasks following the QET intervention. The transfer of skills observed in the RTA, go/no-go, and MSL-aiming tasks underscores the potentially broader implications of the QET programme in enhancing motor coordination and proficiency.

A potential limitation of this study might be the nonparametric transformation of data that was performed to analyse the effects of the eye fixation training. However, the transformation of the test scores to the ordinal categories was needed with respect to the character of the data and data distribution in the specific sample with ADHD. Second, the findings on the possible effects of specific eye-fixation training have been derived from the tendencies across all the samples without consideration of a detailed clinical psychological profile of each child.

However, the underlying neural mechanisms linking attention, inhibition, fine motor skills, and ADHD are complex and require further investigation. Future neuroimaging studies could provide detailed insights into the neural circuitry changes associated with observed behavioural improvements. In addition, long-term follow-up studies are required to assess the persistence of the observed effects over time.

## Figures and Tables

**Table 1:** Basic demographic and clinical information of the sample.

	QET Group	Control Group
	*n* = 54	*n* = 52
age (years)	10.3 ± 1.1	10.7 ± 0.9
sex (% boys)	74.1	71.2
ADHD-I (*n*)	48	43
ADHD-C (*n*)	6	9
medication (*n*)	9	6

Note. ADHD-I: inattentive type; ADHD-C: combined type.

**Table 2:** The tasks of the quiet eye visuomotor training.

Training Session	Major Tasks
1st	Throwing the ball against a wall (2 m distance) and catching with two hands after bouncing
2nd	Overarm throwing a beanbag on one of the three vertical openings of the construction (2 m distance)
3rd	Dart throwing from a distance of 2.37 m
4th	Underarm throwing a beanbag on one of the three horizontal openings of the construction (2 m distance)
5th	Throwing a bolas tool to catch on a vertical rung
	**Modifications of the tasks**
	Throwing from different distances and angles to a target
	The change in an alternative target (with the exception of the first session)
	Using a non-preferred hand for throwing
	Different variations in the changes for the aforementioned modifications

**Table 3:** Medians and median absolute deviations for all test variables in the QET and CON groups.

Variable	QET Group		CON Group	
	Pre-Test	Post-Test	Pre-Test	Post-Test
Reaction test of alertness				
MRT (ms)	356.0 ± 34.1	333.0 ± 52.6	361.0 ± 56.3	354.0 ± 37.8
SDRT (ms)	53.0 ± 19.3	46.0 ± 12.6	55.0 ± 11.9	58.5 ± 20.0
corrR (*n*)	27.0 ± 1.5	28.0 ± 0.0	27.0 ± 1.5	27.0 ± 1.5
incorrR (*n*)	1.0 ± 1.5	0.0 ± 0.0	1.0 ± 0.0	0.5 ± 0.7
GNG inhibition test				
MRT_GNG_ (ms)	415.0 ± 19.3	396.5 ± 14.8	411.0 ± 22.2	394.5 ± 49.7
SDRT_GNG_ (ms)	52.0 ± 5.9	57.5 ± 11.9	51.0 ± 8.9	55.0 ± 13.3
Omission (*n*)	35.0 ± 14.8	23.0 ± 16.3	29.0 ± 14.8	25.0 ± 13.3
Commission (*n*)	7.0 ± 6.7	4.0 ± 3.5	3.0 ± 1.5	7.0 ± 3.0
MLS test: right hand				
Aiming: number of hits	19.0 ± 1.5	20.0 ± 0.0	20.0 ± 0.0	20.0 ± 1.5
Aiming: time of errors (s)	0.04 ± 0.06	0.03 ± 0.04	0.00 ± 0.00	0.06 ± 0.07
Aiming: time (s)	9.98 ± 2.00	10.11 ± 2.25	9.16 ± 1.76	9.56 ± 2.08
Steadiness: time of errors (s)	1.57 ± 1.70	2.27 ± 3.04	0.52 ± 0.77	2.09 ± 2.77
Line tracing: time of errors (s)	4.73 ± 1.69	4.00 ± 1.61	3.72 ± 1.75	3.87 ± 1.39
Line tracing: time (s)	22.80 ± 11.02	22.84 ± 10.73	20.92 ± 7.16	19.30 ± 9.76
Inserting pins: time (s)	55.14 ± 6.83	54.19 ± 6.80	53.16 ± 6.33	54.59 ± 8.27
Tapping: number of hits	164.0 ± 17.8	160.5 ± 18.5	169.0 ± 23.7	168.0 ± 18.5
MLS test: left hand				
Aiming: number of hits	18.0 ± 3.0	18.0 ± 1.5	19.0 ± 3.0	19.0 ± 1.5
Aiming: time of errors (s)	0.14 ± 0.18	0.16 ± 0.16	0.17 ± 0.12	0.13 ± 0.19
Aiming: time (s)	10.35 ± 1.66	10.54 ± 2.33	10.33 ± 2.00	10.42 ± 1.92
Steadiness: time of errors (s)	2.20 ± 2.91	1.59 ± 2.12	4.45 ± 5.72	2.90 ± 4.04
Line tracing: time of errors (s)	4.96 ± 1.93	4.84 ± 1.56	4.78 ± 1.36	4.98 ± 1.88
Line tracing: time (s)	15.00 ± 8.29	18.75 ± 7.87	13.75 ± 7.89	17.61 ± 7.80
Inserting pins: time (s)	58.47 ± 7.89	60.16 ± 5.97	57.66 ± 8.33	57.82 ± 8.14
Tapping: number of hits	141.0 ± 20.8	137.5 ± 20.8	146.0 ± 20.8	138.00 ± 20.8

Note. The absolute deviations from the medians were adjusted by a factor of 1.4826 to ensure asymptotically normal consistency with the standard deviations. Reaction test of alertness: MRT: mean reaction time; SDRT: standard deviation of reaction times; corrR and incorrR: number of correct and incorrect responses, respectively. Go/No-go inhibition test: MRT_GNG_: mean reaction time to go stimuli; SDRT_GNG_: intraindividual standard deviation of RTs to go stimuli.

**Table 4:** Comparison of time (pre-test vs. post-test) effects in the QET and CON groups.

Variable	QET Group		CON Group		Interaction	
	*β*	*p*	*β*	*p*	*β*	*p*
Reaction test of alertness						
MRT	−0.447	0.189	0.304	0.374	−0.751	0.121
SDRT	0.363	0.233	0.278	0.379	0.085	0.846
corrR	0.408	0.177	−0.513	0.111	0.920	0.039 *
incorrR	−0.267	0.402	0.411	0.243	−0.678	0.156
GNG inhibition test						
MRT_GNG_	−0.463	0.170	−0.216	0.517	−.247	0.602
SDRT_GNG_	0.185	0.537	−0.235	0.457	0.420	0.336
Omission	−0.935	0.005 *	0.413	0.193	−1.348	0.004 *
Commission	−0.628	0.038 *	0.463	0.137	−1.090	0.014 *
MLS test: right hand						
Aiming: number of hits	1.187	0.001 *	−0.443	0.227	1.630	0.002 *
Aiming: time of errors	−0.812	0.012 *	0.348	0.267	−1.160	0.011 *
Aiming: time	−0.539	0.101	−1.006	0.009 *	0.468	0.339
Steadiness: time of errors	−0.480	0.114	0.110	0.729	−0.590	0.181
Line tracing: time of errors	−0.484	0.110	−0.261	0.383	−0.223	0.592
Line tracing: time	0.095	0.748	−0.279	0.350	0.374	0.400
Inserting pins: time	−0.089	0.792	0.094	0.784	−0.183	0.704
Tapping: number of hits	−0.023	0.950	0.796	0.037 *	−0.819	0.121
MLS test: left hand						
Aiming: number of hits	0.471	0.128	0.032	0.915	0.438	0.310
Aiming: time of errors	0.597	0.086	0.410	0.261	0.187	0.706
Aiming: time	0.058	0.856	−0.297	0.359	0.355	0.435
Steadiness: time of errors	0.796	0.027 *	0.682	0.050 *	0.114	0.812
Line tracing: time of errors	0.086	0.779	−0.115	0.714	0.201	0.647
Line tracing: time	−0.147	0.656	−0.346	0.292	0.200	0.666
Inserting pins: time	0.033	0.914	−0.286	0.360	0.319	0.466
Tapping: number of hits	0.481	0.208	−0.371	0.378	0.853	0.139

Note. *β* denotes the regression coefficient, see above for its interpretation. * *p* < 0.05. For interpretation of interaction term, note that *β*_interaction_ = *β*_experimental_ − *β*_control_. Reaction test of alertness: MRT: mean reaction time; SDRT: standard deviation of reaction times; corrR and incorrR: number of correct and incorrect responses, respectively. Go/No-go inhibition test: MRT_GNG_: mean reaction time to go stimuli; SDRT_GNG_: intraindividual standard deviation of RTs to go stimuli.

## Data Availability

Data are available on request due to privacy and ethical restrictions. The data presented in this study are available on request from the corresponding author.
